# The systematic approach to faculty development activities for biomedical educators

**DOI:** 10.30476/jamp.2020.84341.1135

**Published:** 2020-07

**Authors:** SATEESH BABU ARJA, SIMI PARAMBAN, KUMAR PONNUSAMY, ABARAHAM RATNA JOSEPH NAYAKANTI, RESHMA FATTEH, SIREESHA BALA ARJA

**Affiliations:** 1 Medical Education Department, School of Medicine, Avalon University, Curacao

**Keywords:** Faculty, Biomedical, Educators, Medical education

## Abstract

**Introduction:**

The term continuing professional development encompasses competencies required to practice the high quality medicine, including medical, managerial, ethical, social, and personal skills, whereas continuing medical education refers only to expanding the knowledge and skills required by physicians. The competencies for basic science faculty identified are management and administration, teaching, assessments, curriculum development, and research. This study aimed to evaluate the outcomes of faculty development initiatives at Avalon University School of Medicine and examine the optimal approach to faculty development activities.

**Methods:**

This is a survey-based quantitative study. A cross-sectional survey was conducted after implementing the faculty development activities. We took thirteen basic science faculty members as a unit and recruited them for different faculty development activities from 2015. Faculty members were involved in various faculty development courses, workshops, and training sessions. A survey was conducted among faculty members using a questionnaire on the Likert scale to identify if there are any increased knowledge or skills on teaching and assessment methods, educational scholarship, and scholarly activities after implementing faculty development initiatives. The faculty responses were tabulated and quantified in the Excel sheet and analyzed by SPSS software.

**Results:**

All thirteen faculty members responded to the questionnaire (100% response rate). There was an increased self-reported knowledge and skills of faculty members. 70% of the faculty agreed that they are able to get involved in designing their course learning objectives. 100% of the faculty were aware of different teaching methods, and 93% of them were implementing different types of teaching methods, including small group discussions, flipped classrooms, standardized patient-based teaching, and problem-based learning. 100% of the faculty were aware of different assessment methods and implementing them. There were self-reported and observed behavioral changes.

**Conclusions:**

Faculty development activities at Avalon University School of Medicine have shown to be effective. At larger institutions, the department chair can lead the faculty development activities.

## Introduction

In some countries, the Continuing Professional Development (CPD) is mandatory, and in other countries it is voluntary. A good number of countries are introducing revalidation ( 1 ) and relicensure ( 2 , 3 ). The term CPD encompasses competencies required to practice high quality of medicine including medical, managerial, ethical, social, and personal skills ( 4 ), whereas Continuing Medical Education (CME) refers only to expanding the knowledge and skills required by physicians. 

Schostak et al. ( 5 ) (2010) stated that *‘CPD is valued and is seen as effective when it addresses the needs of individual clinicians, the populations they serve and organization within which they work.’* One of the reviews conducted for the UK Academy of Medical Royal Colleges and the Central Medical Council reached the same conclusion ( 5 ). Such a conclusion is also in alignment with the Swedish Medical Association’s four-step process ( 6 ). The four-step process includes analyzing the need and starting the process, developing supporting functions, collaborating, and following up and evaluating. Professor Grant, in her book entitled *“The good CPD guide”* ( 7 ) also identified the four steps in good CPD approach for clinicians including to identify what to learn, plan how to learn and document, learn, and use the learning and show its effect. 

The academic competencies for medical faculty include leadership, administration, teaching, curriculum development, research, medical informatics, care management, and multiculturalism ( 8 ) and teacher training should be focused on these competencies. Four core values of the medical educators and medical faculty involved in teaching are learner engagement, learner-centeredness, adaptability, and self-reflection, which encompasses six competencies: medical (or content) knowledge, learner-centeredness, interpersonal and communication skills, professionalism and role modeling, practice-based reflection, and systems-based practice. Malathi et al. (2011) also identified four specialized competencies for the medical faculty with additional programmatic roles: program design/implementation, evaluation/scholarship, leadership, and mentorship ( 9 ). 

A systematic review of faculty development initiatives designed to enhance teaching effectiveness entitled *“A 10-year update: BEME Guide No. 40”* ( 10 ) indicated that satisfaction rates were high for faculty development activities. It is also notified that there is an increased gain in knowledge and skills. The authors also reported the changes in self-reported and observed behaviors. However, most of the studies included in this systematic review were the majority of faculty development interventions targeting the practicing clinicians ( 10 ).

Even though in some of the studies ( 8 , 9 ), the competencies like teaching, curriculum development, research, and professionalism are identified as the required competencies for medical faculty, there is lack of research evidence in guiding the approach to the faculty development activities to attain these competencies for medical teachers, especially for basic sciences faculty. This study aimed to determine the optimal approach to the faculty training and faculty development activities of basic science faculty and to examine if the four-step approach ( 7 ) of Grant is applicable to train the teaching medical faculty not only the clinicians. This study also intended to examine the effectiveness of faculty development initiatives at Avalon University School of Medicine (AUSOM). 

## Methods

This quantitative and cross-sectional survey was conducted after implementing the faculty development activities. Faculty development activities were implemented from 2015 to 2019. The outcomes were assessed after implementing the faculty development activities. We used Kirkpatrick and Kirkpatrick’s model (2006) ( 11 ) for evaluating faculty development programs; it describes four levels of outcome: learners’ reaction to the learning activities/faculty development activities (feedback); learning (changes in knowledge and skills); behavior (either self-reported behavioral changes or observed behaviors); and results (changes at the level of the learner and the organization). We were able to evaluate the outcomes at three levels of learning, behavioral changes, and results. The survey was used to gather data from faculty members if there was a self-reported increase in knowledge and skills related to curriculum development, teaching methods, and assessment methods, which reflects the “learning” level of the Kirk Patrick model. The other data gathered were the number of faculty that received different educational fellowships and provided substantial evidence supporting the self-reported increase in skills. Either self-reported behavioral changes or observed behavioral changes were included in the “behavioral” level of Kirk Patrick’s model of evaluation. We noticed some changes at the “results” level, too. 

After implementing the faculty development activities, a survey was conducted in August 2019, to assess the effectiveness of faculty development activities. The survey questionnaire helped to evaluate the faculty development activities against the three levels of the Kirkpatrick’s model of evaluation, including learning, behavior, and results. 

The following questions were used for evaluating the “learning” level of Kirkpatrick evaluation.

- If the faculty are aware of different teaching methods including lectures, small group discussions, flipped classrooms, and hands-on courses including labs and standardized patient-based teaching.
- If the faculty know or understand the learning theory or rationale behind each teaching method that they are using.
- If they are aware of different assessment methods like formative and summative assessments, including MCQs, short answer questions, lab/oral exams, flowcharts/worksheets, and SP-based assessment.
- If the faculty are aware of Bloom’s taxonomy and Miller’s learning Pyramid.
- If the faculty understand the rationale behind using each assessment, including the level of Miller’s learning pyramid for the assessments that they are using.
- Following Questions were used for evaluating the “behavioral” level of Kirkpatrick’s evaluation.
- If the faculty develop or write their own course learning objectives using Bloom’s taxonomy.
- The faculty use different teaching methods including lectures, small group discussions/ flipped classrooms, and hands-on including labs and standardized patient-based teaching.
- If the faculty use different assessment methods like formative and summative assessments, including MCQs, short answer questions, lab/oral exams, flowcharts/worksheets, and SP-based assessments.
- If the faculty participate in or are aware of standardization of examinations.

Questions like if the faculty are involved in scholarly work, especially educational scholarship and the number of educational fellowships they achieved, helped in evaluating the “results” of Kirkpatrick’s evaluation. 

The survey questionnaire was developed based on different competencies, including curriculum development, teaching and assessment methods, and educational scholarship and research. The survey questions were designed to include different domains in designing the course/module, teaching methods, assessment and feedback to learners, and educational scholarship and research representing different competencies mentioned above. The faculty senate reviewed the survey questionnaire for validation. Three faculty members responded to the survey questionnaire as pilot testing and after that it was administered to the rest of the faculty members. The responses were gathered on the Likert scale ranging from one to five; one was strongly disagree, two disagree, three neutral/no opinion, four agree, and five strongly agree. Also, we observed the participation of basic science faculty in various teaching and assessment methods using course evaluations, and their involvement in the curriculum development and developing learning modules and course learning objectives. 

Herewith, the approach to the faculty development activities at our school of medicine is described and the four steps of Grant identified in the process. 

### *Identify what to learn*

The self-study and evaluation conducted in the years 2015 and 2016 at AUSOM showed that there was a requirement for the faculty members to be trained or updated in various teaching methods. It was also determined that the faculty need to understand the modern techniques of assessments, including different types of formative and summative assessments. There was a requirement on understanding blueprinting or standard setting. The board of trustees and the higher academic leadership, including the deans, were determined to correct these issues. The academic faculty needed more training on knowledge about curriculum development and assessments. The School had attempted to encourage scholarly achievement by providing rewards for publishing scientific research. However, this was at a very early and basic stage, and there was a need for guidance in researching medical education in particular. The administration also talked to the senior faculty members to determine which development activities were required. With the common consensus among higher academic leadership, administration, and faculty members, it was decided to develop the faculty development activities and invest resources in the faculty development activities to enhance the competencies like teaching, curriculum development, assessments, evaluation, medical education research, and management and leadership. 

### *Plan how to learn and documentation*

Personal development planning is the process of creating an action plan based on awareness, values, reflection, goal setting, and planning for personal development within the context of a career, education, relationship, or self-improvement ( 12 ). The executive dean’s office was determined to track the faculty development activities for basic sciences faculty. This office developed an individualized plan for each faculty member. The plan was developed only in consultation with the faculty member as any CPD system must allow the individual doctor to learn as that physician wishes. We enabled the same method for faculty development activities.

As Schostak et al. ( 5 ) pointed out, ‘the literature stated that there was no “single, singular or correct way of doing CPD” and that the content, context, and processes chosen were going to depend upon spheres of practice, learning styles, and personal preferences.’ Various faculty development activities were identified depending upon what to be learned and the resources available as there was a need for multiple faculty development activities for different competencies. The executive dean and senior academic leaders were responsible for determining suitable faculty development activities. Now, this responsibility is taken by the Medical Education Unit at Avalon University. The executive dean’s office was responsible for securing the budget and resources required for the faculty development activities. The associate dean of basic sciences, who is the manager for basic science program, allowed and adjusted the responsibilities and schedules of these basic sciences faculty members so that they could complete their faculty development activities. 

### *Learning activities*

We had the faculty development activities in curriculum design and development, curriculum evaluation, methods of instruction, program evaluation, research methodology, leadership and management, and student assessment. 

We took thirteen basic science faculty members as a unit and enrolled these faculty members in different faculty development activities from the year 2015 ( 13 ). A total of nine faculty members were enrolled and completed the Essential Skills in Medical Education (ESME) course offered by the International Association of Medical Education in Europe (AMEE). This course has focused modules on the role of trainer/teacher in health care professions, learning outcomes/competencies, curriculum development and implementation, feedback, activity, individualization, and relevance (FAIR) principles, teachers Toolkit (large group teaching versus small group discussions), and assessments ( 14 ). 

Three faculty members were enrolled and completed the ESME-assessments offered by AMEE which has focused modules on principles of assessment: validity, reliability, and blueprinting, tests of knowledge, OSCE, workplace-based assessments, standard setting of examination procedures, and quality assurance of assessment ( 15 ). Three faculty members were enrolled and completed leadership for sustainability in uncertain times course offered by AMEE which has focused modules on leading at the edge of uncertainty, working better together, finding the fix that fits, and making the good better.

Besides, six faculty members were enrolled and completed the Introduction to the Principles and Practice of Clinical Research (IPPCR) course offered by the National Institute of Health (NIH). A faculty member who was well versed with medical education research held a workshop and trained all faculty members in medical education research, both quantitative and qualitative research methods. And also, one faculty member was enrolled in Masters in Health Professions Education (MHPE) offered by Foundation for Advancement of International Medical Education and Research (FAIMER), Center for Medical Education in the Context, and Keele University. The faculty member who was enrolled in the Master’s program held the workshops for faculty members on Miller’s learning Pyramid, Bloom’s taxonomy, formative and summative assessment methods, blueprinting, multiple choice questions item analysis, and standardized examination procedures as series of workshops. 

This is a survey-based quantitative study. The faculty development activities were carried out over the years from 2015 to 2019 and is still continuing. A cross-sectional survey was conducted among the faculty members in August 2019 to identify the perceptions of faculty members and find out how they were equipped with various teaching and assessment methods, educational scholarship, and scholarly activities. The survey was conducted using the questionnaire on the Likert scale of one to five. One is strongly disagree, two is agree, three is neutral or no opinion, four is agree and five is strongly disagree. Participation in the survey was completely voluntary and anonymous. All faculty members had the right to decline if they decided not to participate in the survey. A total of thirteen faculty members were involved in the study who received a series of faculty development activities. These thirteen faculty members were involved in teaching different biomedical/basic sciences for medical students. They all were at various ranks, including professor, associate professor, and assistant professor. Different responses of faculty members were tabulated and quantified in the Excel sheet and analyzed by SPSS software. Responses were quantified based on the number of faculty who answered each category. For example, if eight faculty members out of thirteen members responded “strongly agree” (five on the Likert scale of one-five) to a question, then the response rate for strongly agree was 62%. If there was any value with a decimal number greater than 5 (0.5), it was rounded to the nearest next number. 

## Results

### *Use the learning and show its effect*

In the results and discussion section, we emphasized the effects of faculty development activities on daily practice of these faculty members in teaching and assessing the students. The survey questionnaire was given to all thirteen basic sciences faculty members and answered by all thirteen faculty members (100% response rate).

The faculty not only applied the learned principles, but also were able to write reflectively and apply them to different fellowships in medical education/education. Five out of thirteen (38%) received the fellowship from the Academy of Medical Educators, UK in all five domains of the academy as they met the professional standards framework set by the academy (Table 1). Two members received associate fellowship offered by AMEE. One faculty member received the fellowship from higher education academy (FHEA), UK and another one received a senior fellowship from the higher education academy (SFHEA). In addition, three faculty members were awarded the fellowship by International Association of Medical Science Educators (IAMSE).

## Discussion

Faculty members were enrolled in faculty development activities and completed them, but they were reflectively practicing the learned principles in curriculum development, teaching, and assessment methods. Many faculty members reported an increase in knowledge and skills related to educational processes, teaching methods, and assessment methods, which is in correlation with Steinert et al.’s (2016) systematic review of faculty development initiatives ( 10 ), which reflects the “learning” level of Kirkpatrick’s model ( 11 ). It is shown that the faculty members understand different teaching methods. It is also interesting to note that the faculty members understand (92%) the educational theory or rationale behind various teaching methods. It is worth noting that 100% of the faculty members are aware of different assessment methods like formative and summative assessments, including MCQs, short answer questions, lab/oral exams, flowcharts/worksheets, and SP-based assessments. Eighty five percent of the faculty members understand the rationale behind using each assessment, including the level of Miller’s learning pyramid for the assessments except two faculty members out of thirteen faculty members who responded to the survey questionnaire. 

**Table 1:** Survey questionnaire responses analysis

Item/question	Strongly disagree	Disagree	Neutral/no opinion	Agree	Strongly agree
	1	2	3	4	5
Do you develop or write your own course learning objectives using Bloom’s taxonomy? Course/module designing and development.	8%		23%	8%	62%
Are you aware of different teaching methods including lectures, small group discussions/ flipped classroom, and hands-on including labs and standardized patient-based teaching?					100%
Do you use different teaching methods including lectures, small group discussions/ flipped classroom, and hands-on including labs and standardized patient-based teaching?	8%			31%	62%
Do you know or do you understand the learning theory or rationale behind each teaching method that you are using?			8%	23%	69%
Are you aware of different assessment methods like formative and summative assessments, including MCQs, short answer questions, lab/oral exams, flowcharts/worksheets, and SP-based assessments?					100%
Do you use different assessment methods like formative and summative assessments, including MCQs, short answer questions, lab/oral exams, flowcharts/worksheets, and SP-based assessments?				15%	85%
Are you aware of Bloom’s taxonomy and Miller’s learning Pyramid?				31%	69%
Do you understand the rationale behind using each assessment, including the level of Miller’s learning pyramid for the assessments that you are using?	8%		8%	23%	62%
Do you know how to do MCQs item analysis?			8%	23%	69%
Do you participate or aware of standardization of examinations?			8%	15%	77%
Do you involve in scholarly work, especially educational scholarship?		8%	23%	15%	54%

There were reported changes in behavior, either self-reported or observed changes in the behavior, which is also in the same line with Steinert et al.’s (2016) systematic review of faculty development initiatives ( 10 ), which reflects the “behavior” level of Kirkpatrick’s model ( 11 ). Faculty members were actively involved in the curriculum development when we moved from a discipline-based curriculum to an integrated curriculum in 2017 and faculty members wrote the learning objectives for all courses and modules ( 16 ). The faculties (93%) are able to implement different types of teaching methods including small group discussions, PBL, flipped classroom, and student-led seminars. This was evident in course evaluations by students ( 16 ). Faculty members (100%) are applying the principles of formative and summative assessments and implement different assessment methods including but not limited to multiple choice questions, standardized patient-based assessments, concept mapping/flowcharts, laboratory and oral examinations, and short answers. It is also shown that 92% of the faculty either do or understand the standardization of examinations and do know how to do MCQs item analysis.

According to Steinart et al.’s (2016) systematic review of faculty development initiatives, some studies have shown changes in local and national networks ( 10 ) which is reflecting the “results” level of Kirkpatrick’s model ( 11 ). In our study after faculty development activities, the faculty members were able to be a part of international networks like the Academy of Medical Educators, AMEE, Higher Education Academy, and IAMSE and were able to attain the fellowships and associate fellowships. The five domains of the Academy of Medical Educators, UK include designing and planning learning, teaching and facilitating learning, assessment of learning, educational research and scholarship, and educational management and leadership ( 17 ). The professional framework for FHEA has some domains including design and plan learning activities, teach and/or support learning, assess and give feedback to learners, develop effective learning environments and approaches to student support and guidance, and engage in continuing professional development in subjects/disciplines and their pedagogy, incorporating research, scholarship and the evaluation of professional practices ( 18 ). The fellowship of IAMSE requires completion of a project which results in educational scholarship and demonstrates the application of content themes at the home institution (AUSOM) along with completion of ESME course and two-day-long faculty development courses ( 19 ). The one area where the faculty members need improvement is involvement in the scholarly work and participation in educational scholarship. Only 69% is satisfactorily involved in scholarly work and educational scholarship. 

### *Limitations*

One of the limitations identified in this study is the small sample size (12 was the number of faculty when we started in 2015, but now the total basic science faculty involved are 13). This approach can be implemented at larger institutions, too. However, for larger institutions, the head of the department or chair of the department can take responsibility and lead the faculty development activities. The other limitation is faculty attrition. As this approach of faculty development started in 2015 and is now continuing, some faculty left the institution. However, the faculty attrition is very minimal at our school of medicine ( 13 ). And newly joined junior faculty are also enrolled in faculty development courses as required. 

## Conclusion

The approach to faculty development activities at Avalon University School of Medicine has shown to be effective. The four-step approach for clinicians proposed by Grant in her book “The good CPD guide” including identify what to learn, plan how to learn and documentation, learn, and use the learning and show its effect seems to be useful even for biomedical educators. But this study lays the foundation for more studies at larger institutions if Grant’s approach can be valid even for biomedical educators. Even though these faculty development activities are rewarding, we can come across the hurdles. The most crucial obstacle is the availability of the resources, and the institutions should be ready to invest in such activities. We did not come across such a problem as our institute is ready to allocate funds and resources for the faculty development activities. The other problem is that the faculty should be able to buy-in their time and should be prepared to participate in faculty development activities. 

**Take home message**

- The four-step approach proposed by Grant in “The good CPD guide” identify including what to learn, plan how to learn and documentation, learn, and use the learning and show its effect seems to be useful even for basic sciences faculty.
- Faculty’s time and readiness to get involved in professional development activities is the critical factor for faculty development.
- The academic leaders should take measures for the faculty development activities and allocate appropriate budget and resources for faculty development activities.
- At larger institutions, the department chair can lead the faculty development activities.
